# Pain Management in the Elderly: An FDA Safe Use Initiative Expert Panel's View on Preventable Harm Associated with NSAID Therapy

**DOI:** 10.1155/2012/196159

**Published:** 2012-02-14

**Authors:** Robert Taylor, Salma Lemtouni, Karen Weiss, Joseph V. Pergolizzi

**Affiliations:** ^1^NEMA Research Inc., 840-111th Avenue North Suite no. 9, Naples, FL 34108, USA; ^2^FDA Safe Use Initiative, (SUI), CDER, FDA, 11093 New Hampshire Avenue, Silver Spring, MD 20993, USA; ^3^Department of Medicine, John Hopkins University School of Medicine, Baltimore, MD 21205, USA

## Abstract

Optimization of current pain management strategies is necessary in order to reduce medication risks. Promoting patient and healthcare provider education on pain and pain medications is an essential step in reducing inadequate prescribing behaviors and adverse events. In an effort to raise awareness on medication safety, the FDA has launched the Safe Use Initiative program. The program seeks to identify areas with the greatest amount of preventable harm and help promote new methods and practices to reduce medication risks. Since the establishment of the program, FDA's Safe Use initiative staff convened a panel of key opinion leaders throughout the medical community to address pain management in older adults (≥65 years of age). The aim of the expert panel was to focus on areas where significant risk occurs and where potential interventions will be feasible, implementable, and lead to substantial impact. The panel suggested one focus could be the use of NSAIDs for pain management in the elderly.

## 1. Medication Risks

Medication use can result in unwanted adverse reactions that can cause injury and even death. It was documented in 2001 that over 4 million people experienced medication adverse events, which was about 1.5 times the rate observed in 1995 [1]. The Institute of Medicine has estimated that 1.5 million preventable adverse events occur every year [2] and up to 50% could have been prevented by the modification of prescribing methods [3, 4]. Certain risks cannot be avoided, and these include unknown risks that do not manifest during the medication development program and unpreventable known side effect risks, which result even if drug administration is executed optimally, that factor in the risk-benefit evaluation during the regulatory review process of innovative medications. Though these risks are of great concern, proper precaution and prescribing methods cannot circumvent or minimize them [5]. FDA's Safe Use Initiative (SUI) focuses on preventable risks that are significant and amenable to implementable interventions that have potential impact and measurable outcomes.

Identifying, describing, and understanding the root cause of significant preventable risks are essential for reducing harm from medications. FDA's SUI describes four main categories of preventable risks: medication errors, unintended/accidental exposure, intentional misuse/abuse, and drug quality defects [5]. Medication errors can also be broken into subcategories: informational errors in prescribing or by patients/consumers, and procedure and process type errors. Procedure type errors are frequent, especially in hospital settings. It was reported that in the ICU individual patients experienced 1.7 medication errors per day [6]. A review of the literature focusing on hospital practices identified that most medication errors occurred during administration (53%), but they also occurred during prescription (17%), preparation (14%), and transcription (11%) [7]. Though the procedural and process type errors are of concern, the major focus of FDA's SUI is informational errors [5].

## 2. Informational Errors: A Major Focus of the SUI

Informational errors occur during the different stages of the prescribing process and can be a result of the healthcare worker or patient's limited access to the available knowledge on a particular medication. Examples of such events occur when a physician prescribes an NSAID to a patient with known cardiovascular risk factors; patients “overdose” or intake greater than the recommended dosage because approved therapeutic doses did not relieve the symptoms, or when proper professional monitoring of the patient while on medication is either not feasible or not part of the treatment protocol. More attention to education and awareness among patients, caregivers, and healthcare professionals could reduce or eliminate these errors, and methods to promote education will be discussed later.

Since the launch of the SUI in November of 2009, the FDA met with stakeholders and has held workshops in order to identify areas of preventable harm and discuss how to improve current practices to reduce informational errors. One group of experts convened to address preventable harm associated with pain medication in older adults and identified the prescribing practices of NSAIDs as a therapy where medication errors potentially occur. Treating pain in elderly patients is a complex task, requiring a tailored and patient focused approach to prescribing. Because a “one-size-fits-all” approach is neither beneficial nor safe for this population, physicians need to be properly informed about the factors that increase the odds of harm from this class of medications, risk profiles of the different NSAIDs, available prescribing guidelines, and available pain management options. Areas of concern related to NSAID use in the elderly are discussed in order to promote awareness of where changes in the current practice might have an impact.

## 3. Complexity of the Elderly in Pain Management

 The elderly population is normally classified as being greater than 65 years of age. The information provided by the Administration of Aging estimates that by 2030 there will be 72.1 million people over the age of 65 in the United States, which is approximately double what the population was in 2000 [8]. Better lifestyles through proper dieting/exercise and more efficient disease management therapies (e.g., surgical/pharmaceutical technology) have contributed to this population rise. Prescribing pain medications within the elderly population requires the skill of a very knowledgeable physician to navigate through the numerous variables (e.g., physiologic changes, comorbid conditions, multiple medications) that make the elderly population a heterogeneous and complex population to treat [9]. Additionally, the lack of evidence-based practices, training, awareness, and thorough understanding of these variables increase the risk of pain medication safety events in an already complex patient.

 An initial task during one of the workshops was to identify factors impacting the safe use of pain medications in the elderly (Table 1). The pathophysiologic profile of older adults significantly changes with age. Decline in organ function, particularly the renal and hepatic functions, which are critical in the clearance of ingested substances, dictates the pharmacokinetic properties of many drugs [10]. In addition, aging patients can experience changes in body fat and water composition [10, 11]. These changes alter the tissue and plasma distributions of many lipophilic and hydrophilic drugs in ways that predispose patients to adverse effects [10]. Decline in the cognitive function is also an area of concern with aging adults [10]. Impairment of cognitive function can lower medication literacy [12, 13], which has been associated with a decrease in the likelihood of reading prescription leaflets or additional medication information [14, 15], a reduction in medication reconciliation (agreement between physicians and patients on what medications are currently being used) [16], patients misinterpreting dosage directions on drug labels [17], and failure to adhere to verbal counseling by physicians [18].

 Physiologic changes that occur with aging increase the incidence and prevalence of many diseases which directly increases the risk of developing a drug-related disease interaction. In 2009, the Administration of Aging reported that almost all of Medicare beneficiaries had at least one morbid condition while approximately 24% had greater than 4 morbid conditions [20]. Common conditions among the elderly population include diagnosed arthritis, hypertension, heart disease, cancer, diabetes, and sinusitis [20]. Comorbidity is associated with polypharmacy, a phenomena where a single person may be prescribed multiple medications from different doctors or by self-prescribing over-the-counter medications (OTCs) for each medical condition present [21]. A general list of medications that many elderly may be on include cardiovascular drugs (thiazide diuretics, (ACE) inhibitors, beta-blockers); antidiabetic drugs (sulfonylureas, thiazolidinediones, and metformin); fluticasone/salmeterol, tiotropium, albuterol, bronchodilators and steroid inhaler, nonsedating antihistamines, and steroid nasal sprays [22]. Depending on the definition of polypharmacy or type of study conducted, anywhere from 13 to 92% of the geriatric population is on more than one drug [22]. Other studies indicate 80% of patients over the age of 65 years have at least one chronic condition while 50% have more than one; this same group uses between 2 to 6 prescribed medications and 1 to 3.4 nonprescription medications on a regular basis [23, 24]. As the number of medications that are taken increases, the risk of adverse drug reactions increases [24]. Patients taking two drugs concurrently have a 13% risk of adverse drug interactions, those taking four drugs have a risk of 38%, and those taking seven or more drugs have a risk of up to 82% [25]. Another issue that arises from polypharmacy is that it becomes difficult for multiple physicians, pharmacists, and even the patients themselves to keep track of all the medications, thus increasing the odds of drug-drug interactions [26].

 Understanding, preventing, monitoring, and treating adverse events in the elderly are difficult tasks. Whether it is just aging in itself and/or the physiologic changes associated with aging, the elderly population is at increased risk of experiencing more adverse events [27–29]. In the 2011 DAWN report, it was estimated that in 2008 more than 31% of people 65 and older were hospitalized due to a medication adverse event and that patients older than 50 represented 51.5% of all hospitalizations related to adverse events [30]. The challenge is that often it is difficult to discern between the aging process itself, associated comorbidities, and an adverse event that is secondary to drug therapies. Some symptoms such as lethargy, confusion, lightheadedness, falls, constipation, and depression that are observed as side effects of a number of medications are often experienced in elderly patients not on any particular drug [31, 32]. In the dominant climate of evidence-based medicine, there is a general unease regarding how medications affect the elderly population. Even though enrollment of older adults in clinical trials has been occurring since the 1980s, the representation of the “complex” elderly population is limited [33], rendering optimal evaluation of the benefits and risks of any medication to be a challenging task. Complex elderly people are typically aged 75 and older, have multiple concomitant illnesses, take multiple medications, and have some level of functional decline, cognitive impairment, and limited social support [33]. In addition to homogenous selection process among the elderly for the clinical trials, most clinical trials also require “homogenous” treatment plans. Therefore, it is also hard to evaluate adverse events associated with drug-drug interactions.

## 4. Physician Barriers in NSAID Pain Management

The nonsteroidal anti-inflammatory drugs have been a mainstay option for chronic pain management for many years. Adverse side effects associated with NSAIDs including gastrointestinal, cardiovascular, renal, and hematological, have been known for a long time. However, introduction of new drugs into the marketplace and the continuous stream of new research data have recently called into question the use and prescribing guidelines of NSAIDs in the elderly, especially “complex” elderly patients [34, 35].

 The workshop assembled by FDA's SUI wanted to determine areas in the prescribing process of NSAIDs that may lead to medication errors and potential injury. Optimal pain management and an increased risk of harm may occur due to “physician barriers”, factors that may hinder or prevent a physician from adequately and safely treating a patient (Table 2). Prescribing NSAIDs to any patient, especially the elderly, requires knowledge of individual patient risk factors, the ability to assess the benefits and risks of the NSAID, and the responsibility for educating patients and monitoring for effectiveness and side effects of the prescribed NSAID. A recent report demonstrated that more than 50% of patients were not properly informed by a physician or pharmacist on the side effects associated with Rx or OTC NSAIDs [36]. Recent data show that the majority of physicians studied are unaware of potential complications associated with cardiovascular [37] and gastrointestinal systems [38, 39]. NSAID guidelines have been established to increase physician awareness of the complications associated with NSAID use; however, some physicians either do not recognize or do not adhere to such guidelines [40]. A recent survey of physicians identified six major barriers that affected their use of established NSAID guidelines [41]. The barriers mentioned were as follows: lack of familiarity with the guidelines, perceived limited validity of the guidelines, limited applicability of the guidelines to specific patient populations, clinical inertia, anecdotal experiences, and clinical heuristics [41]. The lack of familiarity was attributed to the overwhelming number of published medical guidelines and difficulties in keeping up to date with new recommendations. In support of this, a search of the literature identified more than 20 different guidelines that mention NSAIDs and the elderly in addition to other highly acclaimed medication risk factor guidelines or tools (Table 3).

In addition to education on NSAIDs, prevention and monitoring mechanisms should also be followed or adhered to appropriately in order to provide additional levels of protection for the patients. For example, in a survey of 615 elderly patients in an outpatient or old age/nursing home setting for the coadministration of an NSAID with a prophylactic product to protect against gastrointestinal side effects, 65.3%, 76.2%, and 42.6% failed to receive necessary prophylaxis in the outpatient, old age, and nursing home settings, respectively [42], even though treatment for such side effects are documented in well-established guidelines put forth by the American Geriatric Society [43]. Another safeguard is the task of proper documentation of medical history. A large French study known as CADEUS (COX-2 inhibitors and NSAIDs: description of users) showed that there was a failure on the part of physicians to document and keep patient records. More than 50% of the medical histories described by the patient were not recorded by the physician [44]. OTC drugs and dietary supplements are often believed to be risk-free [45] and are not asked about or documented. Prescribers need to be aware of the possibility of over-dosing on NSAIDs that might result from prescribing and/or taking OTC medications that contain the same active NSAID ingredient. A meta-analysis of data from case-control studies revealed that the odds ratio (reference point is nonuse of NSAIDs) for experiencing a serious GI complication was 4.9 in patients taking a single NSAID, 10.7 in patients taking two, and 60.0 in patients taking three NSAIDs simultaneously [46].  Table 4 identifies some of the common prescription and OTC NSAID medications in hopes of promoting awareness. In addition, combination of certain medications or herbal additives with prescribed or OTC NSAIDs may intensify or mask the side effects associated with NSAIDs; for example, corticosteroids [47], *ginkgo biloba *[48, 49], warfarin [50], and alcohol [51] can increase the severity of gastrointestinal bleeding or peptic ulcers.

## 5. Patient Barriers in NSAID Pain Management

There are also “patient barriers” that might limit the effectiveness of pain treatment or predispose to greater risk of adverse effects (Table 2). Before taking any type of medication, patients should be fully aware of the risks involved; however, data suggest that current patient education on NSAIDs, in particular side effects and how to manage them, is not adequate [36, 52, 53]. Another study that analyzed 807 NSAID users reported that 54% did not know the side effects associated with NSAIDs; additionally, it reported that 33% believed prescription NSAIDs were safer, 32% believed OTC NSAIDs were safer, 20% believed there was no difference, and 15% did not know. Sixty percent and 29% of exclusive OTC NSAID users were neither aware or did not believe they were at risk of side effects from NSAIDs, respectively [54].

In addition to being ill-informed on the side effects of taking a single NSAID, patients are also unaware of the consequences of taking multiple NSAIDs or taking NSAIDs for long periods of time. A recent report analyzed responses from rheumatologic pain patients on their knowledge of prescribed NSAID and OTC NSAID risks [55]. Forty-nine percent knew that taking multiple NSAIDs increased the risks of side effects, 41% were uncertain, and 10% did not believe the claim [55]. Some of the reasons for taking multiple doses of NSAIDs include seeking more or faster relief, experiencing no relief with the recommended dose, or a result of doctor's suggestion [54]. In addition, patients are sometimes unaware of or misinformed by practitioners about other medications that contain NSAIDs, like cold or flu OTC medicines [54]. In order to promote awareness, some of the common medications containing NSAIDs are listed in Table 4.

## 6. Interventions

**Figure 1:**
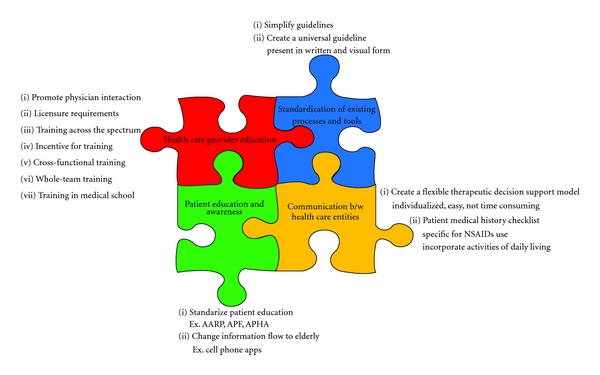
Interventions for optimizing prescribing practices of NSAIDs in the elderly.

 In addition to identifying areas of concern during NSAID treatment of the elderly, the workshop participants brainstormed possible ways to address some of the issues mentioned throughout this paper. These interventions are solely presented as suggestions and are meant to stimulate further discussion within the health care community. For simplicity, the information presented throughout this paper can be divided into 4 categories: (1) health care provider training/knowledge/education/awareness; (2) patient education/awareness; (3) optimization and use of standardized processes/mechanisms/tools; (4) communication between the healthcare entities involved and care coordination (Figure 1). Working on and improving these areas can be an excellent opportunity to mitigate preventable adverse side effects. In order to improve health care provider education, some ideas include licensure requirements, specialty training in geriatric pain management, and incorporation of training in medical schools. Currently there are many guidelines that outline prescribing methods for NSAIDs in the elderly (Table 3). However, healthcare providers can become overwhelmed and confused regarding which guidelines are more appropriate. A suggestion to correct for this would be to simplify and present one unifying document either in a written and/or visual form. Suggestions to improve patient education and awareness include standardizing patient education materials and/or incorporating new technology to increase information flow to and assimilation by the elderly. Underutilized technology may include visual aids, which can take the form of videos played in doctor's office waiting rooms, podcasts for computers or cell phones, even YouTube videos. Other suggestions may be to incentivize communication between healthcare providers and patients, for example, in primary care offices where nurses take on a large role disseminating medical information. Finally, opening up the lines of communication between all healthcare entities would allow for a more patient-focused treatment. Additional intervention ideas included establishment of a therapeutic decision support model or even a required checklist designed to cover all points of a patient's medical history.

## 7. Conclusion

 Medication errors can have a serious public health impact and be costly. The Safe Use Initiative (SUI) is a nonregulatory effort within FDA; one of its focus points is to identify areas where medication errors can be prevented. Through collaboration with key stakeholders, the SUI hopes to raise awareness of these issues and help the health care community make changes. As a result of the combined efforts of SUI and its partners, a number of key areas were identified as worth addressing in order to optimize pain therapy. Healthcare provider education on NSAID use is necessary for the protection of a patient's wellbeing. Improving physician (and other healthcare providers) adherence to NSAID guidelines and enhancing understanding of the pharmacology of NSAIDs in the geriatric population is an essential step to reducing medication errors. Finding new and innovative ways to promote education and awareness should be a task which the entire medical community addresses.

## Figures and Tables

**Table 1:** Factors that affect prescribing methods in elderly patients.

General factors	
(1) “One-size-fits-all” approach not applicable	
(a) All geriatric patients *≠*	
(2) Physiology	
(a) Renal decline	
(b) Hepatic decline	
(c) Body muscle/fat composition change	
(3) Perception of pain varies	
(4) Cognitive impairment	
(a) Medication literacy decline	
(b) Confusion about what, when, and how much	
medication to take	
(5) Comorbidities	
(a) Interactions with medications	
(b) Require multiple medications	
(6) Polypharmacy	
(a) Confusion about what, when, and how much	
medication to take	
(b) Overdosing	
(c) Drug-drug interactions	
(7) Age-related factors interactions with medication	
(a) Increased susceptibility	
(b) Atypical presentation	
(c) Delayed/lack of detection	
(8) Evidence-based practices	
(a) Lack of a roadmap to optimally manage pain	
(9) Multiplicity of providers and lack of team approach to	
managing health conditions in the elderly	
(a) Multiple physicians prescribing	
(b) Pharmacists	
(c) Self/consumer	
(d) Insurance companies	
(10) Cost/coverage of medications	

**Table 2:** Barriers created during NSAID therapy.

Physician barriers	Patient barriers
(1) Limited awareness of or failure to adhere to available NSAIDs Guidelines	(1) Barriers to patient education regarding the proper use of medications
(a) Too many guidelines	(a) Power of TV ads on decision for what medications to take
(b) Confusion about which guidelines are applicable to what segment of the elderly population	(b) Limited awareness of the risks associated with medications
(2) Barriers to using available and up-to-date knowledge on NSAIDs	(2) Failure to objectively, properly, and safely alleviate pain
	(a) Prevalence and intensity of pain sometimes clouds patients' reasoning and leads to unsafe medication intake behavior
(3) Inability of primary health care providers to pursue education/training on how to adjust some of the medical practice aspects to the geriatric population	(3) Economic factors
	(a) Cost of copayments sometimes deter from seeking professional and appropriate prescriptions
(4) Barriers to optimally manage patient's pain which leads the patient to seek other sources of alleviating it	(4) Misperception about the safety of OTC medications
	(a) Risk of overdosing, drug-drug interactions, and chronic use
	(b) Failure to recognize or legitimize the tell-tale signs of adverse events
(5) Limited physician-patient interchange regarding proper use and what to expect	(5) Limited awareness of NSAID sources
	(a) OTC
	(b) Prescription
(6) Absence of systematic screening, monitoring, and educational procedures for prevention	
(a) Identify patients at high risk of renal impairment, GI bleed, cardiovascular side effects, and other AEs and manage them appropriately	
(b) Ask/document OTC use including NSAIDs and dietary supplements	
(c) Warn patients about specific NSAID serious adverse events	
(d) Make patient aware of medications that interact with NSAIDs	
(e) Educate patient about the risk of long-term use of NSAIDs	
(i) Ex. patients at high risk of CVD	
(f) Prescribe prophylactic measures	
(i) Ex. proton pump inhibitors	
(7) Barriers to the use of new technological tools to improve prescribing	
(8) Limited awareness of and evidence-based practice for nonpharmacologic approaches to pain reduction	

**Table 3:** List of current guidelines containing NSAIDs and/or elderly.


(1) Agency for Healthcare Research and Quality (AHRQ)
(2) American Academy of Family Physicians
(3) American Academy of Orthopaedic Surgeons
(4) American College of Gastroenterology
(5) American College of Physicians
(6) American College of Rheumatology
(7) American Geriatrics Society
(8) American Heart Association
(9) American Pain Society
(10) American Society of Anesthesiologists
(11) Institute for Clinical Systems Improvement
(12) Institute for Clinical Systems Improvement
(13) National Headache Foundation
(14) National Kidney Foundation
(15) Osteoarthritis Research Society International (OARSI)
(16) US Headache Consortium
(17) Beers List*
(18) HEIDIS*
(19) STOPP and START*
(20) McLeod*
(21) IPET*
(22) ACOVE*

*Tools to identify potential inappropriate medications.

**Table 4:** List of Rx and OTC NSAIDs [19]: *some items may no longer be currently marketed in the US and current list may not be exhaustive. Data is from 2009.

	Common medications containing aspirin	
Alka Seltzer	Excedrin Extra-Strength Analgesic	Panasal
Anacin	Tablets and Caplets	Percodan Tablets
Arthritis Pain Formulav	Excedrin Migraine	Persistan
Arthritis Foundation Pain Reliever	Fiogesic	Pravigard
ASA Enseals	Fiorgen PF	Rhinocaps
Arthritis Strength Bufferin	Fiorinal (most formulations)	Robaxisal Tablets
Analgesic Caplets	Fiortal	Sine-Off Sinus Medicine Tablets-
ASA Suppositories	Gelpirin	Aspirin Formula
Ascriptin	Genprin	Roxiprim
A/D	Gensan	Saleto
Aspergum	Heartline	Salocol
Asprimox	Headrin	Sodol
Axotal	Isollyl	Soma Compound Tablets
Azdone	Lanoprinal	Soma Compound with
Bayer (most formulations)	Lortab ASA Tablets	Codeine Tablets
BC Powder and Cold	Magnaprin	St. Joseph Adult Chewable Aspirin
Bufferin (most formulations)	Maximum Strength Arthritis Pain	Supac
Buffets II	Formula By the Makers of	Suprin
Buffex	Anacin Analgesic	Synalgos -DC Capsules
Cama Arthritis Pain Reliever	Marnal	Tenol-Plus
COPE	Micrainin	Trigesic
Dasin	Midol	Tri-pain
Darvon Compound 65	Momentum	Talwin Compound
Dolprin no. 3	Norgesic Forte (most formulations)	UN-aspirin
Easprin	Aspirin Norwich Regular Strength	Ursinus
Ecotrin (most formulations)	Aspirin	Vanquish Analgesic Caplets
Empirin Aspirin (most formulations)	PAC Analgesic Tablets	Wesprin Buffered
Epromate	Pain Reliever Tablets	Zee-Seltzer
Equagesic Tablets	Orphengesic	ZORprin
Equazine	Painaid	

	Common medications containing an NSAID	

Actron Caplets	Flurbiprofen	Motrin IB
Advil	Genpril	Nabumetone
Advil Migraine	Ibuprin	Nalfon
Advil Cold and Sinus	Ibuprofen	Naproxen
Aleve	Indomethacin	Naprosyn
Altran	Indocin	Nuprin
Anaprox DS	Ketoprofen	Orudis KT
Ansaid	Ketorolac	Oxaprozin
Arthrotec	Lodine	PediaCare Fever
Bayer Select Pain Relief	Meclofenamate	Piroxicam
Formula Caplets	Mefenamic Acid	Ponstel
Children's Motrin	Meloxicam	Relafen
ClinorilV	Menadol	Saleto 200
Daypro	Midol IB	Sulindac
Diclofenac	Mobic	Toradol
Etodolac		Voltaren
Feldene		
Fenoprofen		
